# Quantitative Analysis of Pb in Soil Using Laser-Induced Breakdown Spectroscopy Based on Signal Enhancement of Conductive Materials

**DOI:** 10.3390/molecules29153699

**Published:** 2024-08-05

**Authors:** Shefeng Li, Qi Zheng, Xiaodan Liu, Peng Liu, Long Yu

**Affiliations:** 1 School of Chemical and Environmental Engineering, Wuhan Polytechnic University, Wuhan 430023, China; lishefeng@whpu.edu.cn (S.L.); zhengqi0310@163.com (Q.Z.); 2 College of Food Science and Engineering, Wuhan Polytechnic University, Wuhan 430023, China; 3 Beijing Construction Engineering Group Environmental Remediation Co., Ltd., Beijing 100015, China; liupeng@bceer.com; 4 Wuhan Regen Environmental Remediation Co., Ltd., Wuhan 430073, China; yulong@rjrem.com

**Keywords:** soil, Pb, laser-induced breakdown spectroscopy, conductive materials

## Abstract

Studying efficient and accurate soil heavy-metal detection technology is of great significance to establishing a modern system for monitoring soil pollution, early warning and risk assessment, which contributes to the continuous improvement of soil quality and the assurance of food safety. Laser-induced breakdown spectroscopy (LIBS) is considered to be an emerging and effective tool for heavy-metal detection, compared with traditional detection technologies. Limited by the soil matrix effect, the LIBS signal of target elements for soil heavy-metal detection is prone to interference, thereby compromising the accuracy of quantitative detection. Thus, a series of signal-enhancement methods are investigated. This study aims to explore the effect of conductive materials of NaCl and graphite on the quantitative detection of lead (Pb) in soil using LIBS, seeking to find a reliable signal-enhancement method of LIBS for the determination of soil heavy-metal elements. The impact of the addition amount of NaCl and graphite on spectral intensity and parameters, including the signal-to-background ratio (SBR), signal-to-noise ratio (SNR), and relative standard deviation (RSD), were investigated, and the mechanism of signal enhancement by NaCl and graphite based on the analysis of the three-dimensional profile data of ablation craters and plasma parameters (plasmatemperature and electron density) were explored. Univariate and multivariate quantitative analysis models including partial least-squares regression (PLSR), least-squares support vector machine (LS-SVM), and extreme learning machine (ELM) were developed for the quantitative detection of Pb in soil with the optimal amount of NaCl and graphite, and the performance of the models was further compared. The PLSR model with the optimal amount of graphite obtained the best prediction performance, with an Rp that reached 0.994. In addition, among the three spectral lines of Pb, the univariate model of Pb I 405.78 nm showed the best prediction performance, with an Rp of 0.984 and the lowest LOD of 26.142 mg/kg. The overall results indicated that the LIBS signal-enhancement method based on conductive materials combined with appropriate chemometric methods could be a potential tool for the accurate quantitative detection of Pb in soil and could provide a reference for environmental monitoring.

## 1. Introduction

Soil is the cornerstone of ecosystem balance and agricultural production, with its health status being directly linked to ecological security, food safety, and human health [1,2]. However, with the rapid development and construction of society, the scale of industries and cities has gradually expanded. The large-scale use of industrial materials and the large-scale discharge of domestic sewage have caused serious soil heavy-metal pollution. Lead (Pb), a quintessential heavy metal, has emerged as a global environmental contaminant due to its persistence, bioaccumulation potential, and long-range transport capacity in the environment [3]. Excessive Pb in soil can change its physicochemical properties, affect soil functions and the growth of animals and plants, and enter the human body through the food chain, thereby posing risks to human health [4,5,6]. Consequently, the rapid and accurate quantitative detection of Pb content in soil is imperative for the surveillance of environmental quality and the safeguarding of food safety. At present, the detection of heavy metals in soil primarily relies on techniques such as atomic absorption spectroscopy (AAS), inductively coupled plasma optical emission spectrometry (ICP-OES), and inductively coupled plasma mass spectrometry (ICP-MS) [7]. However, these conventional analytical methods have several limitations, such as lengthy detection cycles, high costs, and complex operations, which struggle to meet the demands of rapid real-time analysis [8,9].

Laser-induced breakdown spectroscopy (LIBS) is an emerging analytical technique for elemental analysis, utilizing high-energy laser pulses focused onto a sample surface via an optical system to generate a plasma through sample ablation [10]. Both qualitative and quantitative elemental analysis can be realized by analyzing characteristic spectral lines emitted by the plasma, which correspond to specific elements. With the advantage of straightforward sample preparation, fast analytical speed from seconds to minutes [11,12,13], simultaneous detection of multiple elements, real-time in-situ analysis capability, and capability of integration with other methods for multi-dimensional data acquisition [14,15,16], LIBS has been applied across various fields, including agriculture, industry, and environmental monitoring [8,17,18]. However, the quantitative performance of LIBS is limited due to the fact that the target element signals are susceptible to interference by soil matrix effects [19]. Therefore, LIBS signal-enhancement technologies have garnered significant attention among researchers worldwide, including LIBS-instrument modification based on dual-pulse excitation [20,21], spatial confinement [22,23], and magnetic confinement [24,25] and the optimization of sampling environments based on inert gas [26]. Compared with the original LIBS, these methods effectively improve the spectral signal intensity, show good performance for the quantitative detection of soil heavy metals with a correlation coefficient (R) above 0.9, and have a limit of detection (LOD) from 0.132 to 40.6 mg/kg, but they have relatively high costs. In addition, the processing of samples using additives is also one of the most promising techniques for signal enhancement, which has been proved to reduce the matrix effect and improve the coupling efficiency, thereby improving the quantitative analysis performance of LIBS. Xu et al. [27] explored the effect of potassium salt supplemented with halogen group elements on the spectral signal of coal semi-coke powder. With the additives, the relative deviation of the characteristic spectral peak and plasma temperature was significantly reduced, and the quantitative detection accuracy was significantly improved. Goueguel et al. [28] investigated the effect of NaCl concentration on the detection of calcium and potassium in brine by LIBS technology. It was found that the presence of NaCl solution can increase the intensity of characteristic peaks, which may be due to the conductivity of NaCl solution, and increased the plasma temperature, resulting in signal enhancement. Liu et al. [29] mixed gold nanoparticles with metal chelate for LIBS metal-element detection, and the gold nanoparticles improved the spectral intensity and reduced the LOD. Primarily, nanoparticles reduced the ablation threshold of the sample and enhanced the efficiency of laser ablation. Thus, conductive materials could be used to reduce the influence of sample matrix effects and improve detection accuracy. NaCl and graphite, as common and cost-effective conductive materials under certain conditions, exhibit a simple composition that does not interfere with the detection of heavy-metal elements in LIBS analysis. To the best of our knowledge, the quantitative detection of Pb in soil supplemented with NaCl and graphite has not been investigated.

This study aims to investigate the quantitative determination performance of Pb in soil based on the LIBS signal enhancement of NaCl and graphite. The impact of adding varying quantities of NaCl and graphite on spectral intensity and the parameters of Pb were analyzed and compared, and the mechanism of signal enhancement by NaCl and graphite were further explored based on the analysis of the three-dimensional profile data of ablation craters and plasma parameters (plasma, temperature and electron density). Univariate and multivariate quantitative analysis models of Pb in soil with NaCl and graphite were built, and the performance of the models was further compared. The results are expected to provide useful information for signal enhancement in soil samples using conductive materials and help in the accurate quantitative detection of soil heavy metals.

## 2. Results and Discussion

### 2.1. The Influence of Conductive Materials on Spectral Signals

The spectra were collected over the range from 230.55 to 883.77 nm, and a total of 22,036 spectral variables were recorded. Pb I 283.31 nm, Pb I 368.35 nm, and Pb I 405.78 nm are the three primary characteristic lines of the Pb element, according to the National Institute of Standards and Technology (NIST) database. Soil samples with moderate Pb concentrations (1380 mg·kg^−1^) were used as an example for the analysis due to the consistency of spectral trends among samples with various Pb concentrations.

**Figure 1:**
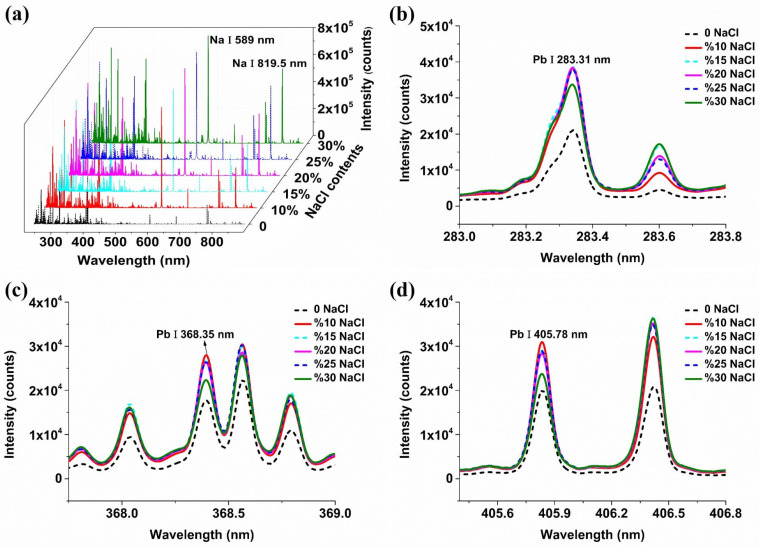
Spectra of soil with different contents of sodium chloride (NaCl). (**a**) Full spectra of soil samples; (**b**) Pb I 283.31 nm spectrum of soil samples; (**c**) Pb I 368.35 nm spectrum of soil samples; (**d**) Pb I 405.78 nm spectrum of soil samples.

Figure 1 shows the average spectra of soil samples with different contents of NaCl. It can be seen from Figure 1a that the overall spectral intensity was significantly improved after adding NaCl, and the intensity of Na I 589 nm and Na I 819.5 nm commonly used in the sodium element was significantly improved. Furthermore, the added content of NaCl has different signal-enhancement effects on different spectral lines. For most elements, the spectral intensity was highest when the NaCl content was 30%. For the characteristic line of Pb I 283.31 nm, the spectral intensity reached the highest when the NaCl content was 15%, but it was close to the intensity of the line when the NaCl content was 10%, 20% and 25%, respectively. When the added content continued to increase to 30%, the spectral intensity decreased significantly. For the characteristic lines of Pb I 368.35 nm and Pb I 405.78 nm, the spectral intensity was the largest when the NaCl content was 10%, and there was a slight decreasing trend with the increase in NaCl content. As the added content rose to 30%, there was a notable reduction in spectral intensity. In general, with the increase in NaCl content, the signal intensity of the three spectral lines of Pb first showed a trend of being approximately unchanged and then significantly decreased, which mainly might be due to the dilution of the target element concentration, indicating that adding a small amount of NaCl can achieve a significant increase in signal intensity for the three spectral lines of Pb.

**Figure 2:**
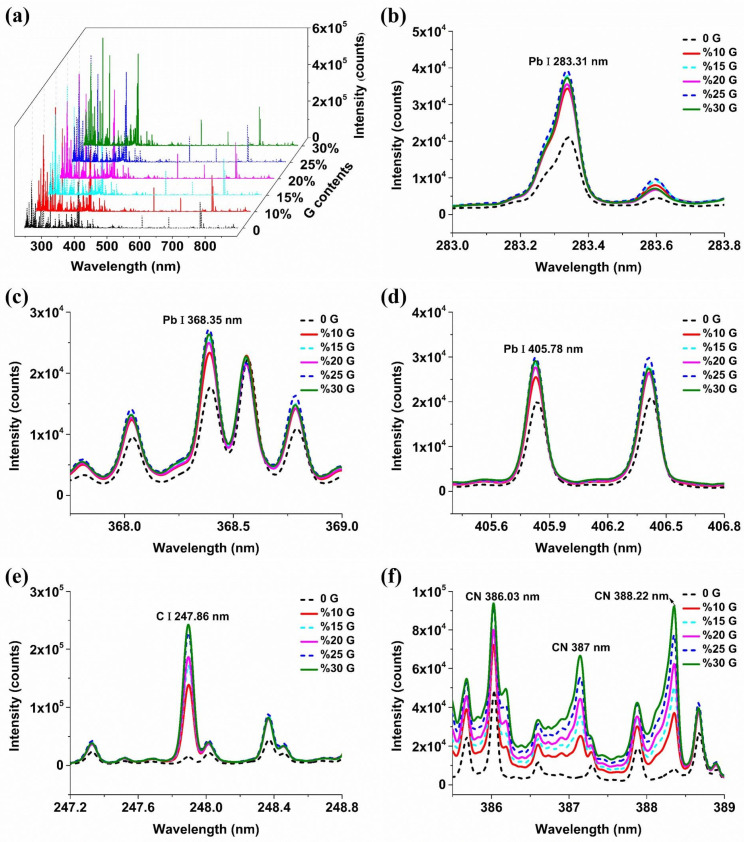
Spectra of soil samples with different contents of graphite. (**a**) Full spectra of soil samples; (**b**) Pb I 283.31 nm spectrum of soil samples; (**c**) Pb I 368.35 nm spectrum of soil samples; (**d**) Pb I 405.78 nm spectrum of soil samples; (**e**) C I 405.78 nm spectrum of soil samples; (**f**) CN 386.03 nm, CN387 nm and CN388.22 nm spectra of soil samples. G means graphite.

Figure 2 shows the average spectra of soil samples with different contents of graphite. It can be seen from Figure 2a that the overall spectral intensity was significantly improved after adding graphite, and the optimal added content of graphite required for the spectral signal enhancement of different elements was different. For most elements, the intensity of the corresponding spectral lines was highest when the graphite content was 30%, which was consistent with the addition of NaCl. For the characteristic line of Pb I 283.31 nm, the intensity of the spectral line was the highest when the graphite content was 25%, and the intensity of the spectral line gradually decreased as the added amount changed to 15%, 30%, 20%, and 10%. For the characteristic lines of Pb I 368.35 nm and Pb I 405.78 nm, the change trend of spectral intensity with the increase in added graphite was consistent. The intensity of the spectral line was the largest when the graphite content was 25%, and gradually decreased as the added content changed from 30% to 10%. In general, different graphite additions have little effect on the signal intensity of the three spectral lines of Pb. The main reason might be that the increase in content graphite will dilute the target elements, and the addition of graphite materials can enhance the intensity of the spectral lines. The two factors worked together to cause the irregular change of spectral intensity with the increase in the added graphite content. This result was different from that found by adding NaCl. However, the intensities of the three spectral lines of Pb were similar by adding NaCl and graphite, and both additive materials can enhance the signal intensities of Pb well. As can be seen from Figure 2e,f, with the increase in graphite content, the intensities of the spectral lines of C I 247.8 nm, CN 386.03 nm, CN 387 nm, and CN 388.22 nm related to graphite gradually increased, which proved that the LIBS spectral intensity had a good response to the element concentration, and the quantitative modeling of elements based on LIBS spectra was reliable.

**Figure 3:**
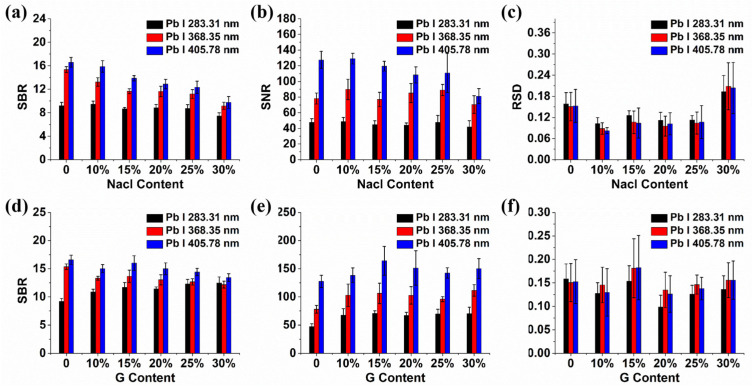
Comparison of Pb spectral-line parameters of soils with different contents of NaCl and graphite. (**a**,**d**) SBR; (**b**,**e**) SNR; (**c**,**f**) RSD. G means graphite.

In the quantitative analysis of elements via LIBS, the detection process is susceptible to interference from systemic noise and the background signals of elements. Key performance indicators, including signal-to-background ratio (SBR), signal-to-noise ratio (SNR), and relative standard deviation (RSD), are pivotal in assessing the system’s stability and its detection [30]. SBR and SNR denote the ratios of the analytical signal to background and noise, respectively, while RSD is quantified as the proportion of a signal’s standard deviation relative to its mean intensity. Typically, higher SNR and SBR values are indicative of better analytical sensitivity, and lower RSD values suggest superior system stability. Consequently, these three parameters were employed to further study the influence of NaCl and graphite on spectral signals. Figure 3 shows the effects of NaCl and graphite on the signal parameters of spectral lines. Regardless of the quantities of graphite and sodium chloride introduced, it is obvious that the SBR of Pb I 405.78 nm remained the most pronounced, while the SBR of Pb I 283.31 nm was comparatively minimal. After adding NaCl, the SBR of the three spectral lines of Pb showed a general downward trend with the increase in NaCl content, in contrast to the trend of spectral intensity. The results indicated that adding NaCl increased the intensity of the background signal, and the enhancement effect was stronger than that on the target spectral line, resulting in a decrease in SBR. It can be seen from Figure 3b that the SNR of Pb I 405.78 nm was greater than that of Pb I 368.35 nm and Pb I 283.31 nm. As the NaCl content increased, the SNR of the three spectral lines of Pb fluctuated continuously, which reached a maximum when the NaCl content was 10%. The result was consistent with the trend of spectral intensity, indicating that adding NaCl had less impact on noise and improved the sensitivity of the spectral line. Figure 3c shows that RSDs of the three spectral lines of Pb were all lower than 0.2, which was a normal result of LIBS detection. As NaCl content increased, the RSDs of the three spectral lines exhibited an initial decrease followed by an increase. At a NaCl content of 10%, the RSD of these three lines reached its minimum, indicating that the spectral signals of Pb were most stable. Considering the spectral intensity and signal parameters, the addition of NaCl to a concentration of 10% yielded optimal results: the near-highest or highest spectral intensity and SNR, the lowest RSD, and a satisfactory SBR. Thus, 10% NaCl content was chosen for preparing soil samples for the accurate quantitative detection of Pb. It can be seen from Figure 3d that before and after adding graphite, the SBR of Pb I 405.78 nm was larger than that of the Pb I 368.35 nm and Pb I 283.31 nm lines before and after adding different amounts of graphite, which was consistent with the result of adding NaCl. The result indicated that Pb I 405.78 nm exhibited superior signal performance compared to the other two lines—a characteristic line that has been leveraged in numerous studies for the quantitative analysis of Pb [31,32]. After adding graphite, the SBR of Pb I 283.31 nm progressively increased, peaking at 30% graphite content. Conversely, the SBR for Pb I 368.35 nm and Pb I 405.78 nm exhibited a fluctuating pattern, and it was at its maximum in the absence of graphite, in accordance with the result of adding NaCl. The results indicated that the addition of graphite intensified the background signal, with a more pronounced effect on the Pb I 283.31 nm line than on Pb I 368.35 nm and Pb I 405.78 nm, resulting in a reduced SBR of the latter two and an increased SBR of Pb I 283.31 nm. Figure 3e illustrates that the SNR for Pb I 405.78 nm surpassed that of Pb I 368.35 nm and Pb I 283.31 nm. As the graphite content increased, the three spectral lines exhibited a biphasic trend, initially rising and then falling before rising again. The SNR of Pb I 283.31 nm and 405.78 nm peaked at a graphite content of 15%. Conversely, the Pb I 368.35 nm line achieved its maximum SNR at 30% graphite content. Notably, the SNR values for the spectral lines remained relatively consistent across different graphite content. Broadly, the RSD of the three spectral lines showed a similar trend with the increase in graphite content; its lowest value was attained at 20% graphite content. The incorporation of an optimal amount of graphite was conducive to enhancing the stability of the Pb spectral signal. Considering the overall impact of graphite addition on the intensity and signal parameters of the three Pb spectral lines, 20% graphite content was employed in the preparation of soil samples for the quantitative determination of Pb.

Upon the addition of 10% NaCl and 20% graphite, the SBR and SNR of the spectral lines remained comparable, corroborating the findings in the signal intensity. The results suggested that the background signal of soil is minimally influenced by these additives. Furthermore, the reduced RSD post-NaCl addition implied that NaCl was more effective in enhancing signal stability than graphite.

### 2.2. Mechanism Analysis of Enhancement of Spectral Signals by Conductive Materials

**Figure 4:**
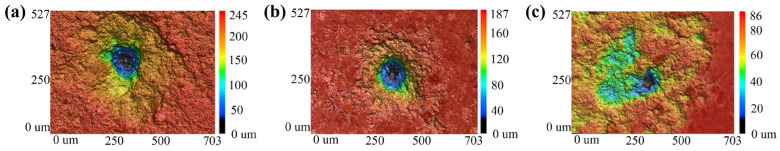
Comparison of the three-dimensional profile of ablation craters of soil samples with different conductive materials. (**a**) NaCl; (**b**) graphite; (**c**) without additives.

The quantitative detection of elements by LIBS relies on plasma produced by the laser ablation of samples, and the inherent characteristics of the sample inevitably affect the formation of ablation craters and the ablation quality of the sample, which were analyzed to investigate the effect of NaCl and graphite on LIBS analysis by a three-dimensional contour scanner (VK-X-1000, Keyence, Osaka, Japan). Figure 4 illustrates the comparison of the morphology of laser-ablated craters in soil samples with the optimal proportion of conductive material. The figure revealed that the addition of conductive materials resulted in distinct inverted conical ablation craters. With 10% NaCl, the mean depth of the crater was 177.181 μm, the mean cross-sectional area was 3.357 × 10^4^ μm^2^, and the mean volume was 2.082 × 10^6^ μm^3^. For 20% graphite, these values were 152.402 μm, 2.349 × 10^4^ μm^2^, and 1.866 × 10^6^ μm^3^, respectively. In the absence of conductive additives, no clear inverted conical crater was observed. Instead, a central borehole due to the ablation effect was observed, with an average depth of 75.630 μm, a cross-sectional area of 7.159 × 10^4^ μm^2^, and a volume of 1.783 × 10^6^ μm^3^. The addition of conductive materials enhanced the ablation efficiency, with the 10% NaCl addition yielding the most pronounced effect. This enhancement might be related to the reduced threshold for laser penetration into soil samples, augmented thermal coupling efficiency between the laser and the sample, and an increased volume of material ablated by the laser. Moreover, NaCl has lower melting (801 °C) and boiling points (1465 °C) compared to graphite, which has a high melting point (3652 °C) and boiling point (3697 °C). Therefore, NaCl offered superior laser absorption and more effective ablation due to its lower phase-transition temperatures.

Plasma temperature and electron density are two important parameters that affect the spectral characteristics of plasma radiation, which can be used to analyze the mechanism of signal enhancement. Specifically, plasma temperature correlates with spectral intensity, while electron density is associated with the linewidth of LIBS spectral lines, and both are pivotal in assessing whether the plasma is in local thermodynamic equilibrium (LTE) [33,34]. Adequate electron density leads to frequent electron collisions, resulting in high plasma excitation, ionization, and electron temperatures, thereby maintaining a state of LTE for plasma that ensures effective quantitative analysis of LIBS. Under the state of LTE, the actual concentration of the element and its corresponding spectral-line signal intensity can be expressed by the following formula:

(1) $$I_{\lambda }^{ij}=C_{s}FA_{ij}\frac{g_{i}}{U_{s}(T)}e^{-E_{i}/k_{B}T}$$

where $I_{\lambda }^{ij}$ is the spectral-line intensity value of the transition radiation between the high-energy level i and the low-energy level j, λ is the wavelength, $C_{s}$ is the concentration of the element, which is the only independent variable in the formula, $A_{ij}$ is the transition probability from the high-energy level to the low-energy level, $g_{i}$ is the statistical weight of the high-energy level, $E_{i}$ is the energy of the high-energy level, $K_{B}$ is the Boltzmann constant, T is the plasma temperature, $U_{s}(T)$ is the temperature-dependent partial differential function, and F is the experimental parameter coefficient, which is related to the optical coupling efficiency, laser ablation efficiency and plasma density.

This is example 2 of an equation:

(2) $$\ln\frac{I_{\lambda }^{ij}}{g_{i}A_{ij}}=-\frac{1}{k_{B}T}E_{i}+\ln\frac{C_{s}F}{U_{s}\left(T\right)}$$

Equation (2) can also be simplified to

(3) $$y_{i}=ax_{i}+b_{s}$$

where $y_{i}=\ln\frac{I_{\lambda }^{ij}}{g_{i}A_{ij}},\;a=-\frac{1}{k_{B}T},{\;x}_{i}=E_{i},{\;b}_{s}=\ln\frac{C_{s}F}{U_{s}\left(T\right)}$. According to the above formula, multiple atomic or ion emission spectral lines of the same element are selected to determine a Boltzmann diagonal line, and the plasma temperature T can be obtained through the slope of the diagonal line.

The electron density of plasma is usually calculated by the Stark broadening method [35,36]. The expressions of Stark broadening and electron density are as follows:

(4) $$∆\lambda_{1/2}=2w\frac{n_{e}}{10^{16}}$$

(5) $$∆\lambda_{1/2}=\sqrt[{2}]{{{∆\lambda_{1}}^{2}-∆\lambda_{2}}^{2}}$$

where $∆\lambda_{1/2}$ represents Stark broadening, $∆\lambda_{1}$ is the experimentally measured full width at half maximum (FWHM) of the spectral line, $∆\lambda_{2}$ is the instrument broadening, and w is the electron or ion collision parameter. The relationship curve between the electron collision coefficient of the elemental spectral line and plasma temperature can be established according to reference [37].

The state of LTE can be evaluated using the plasma temperature and the electron density based on the following formula [38]:

(6) $$n_{e}\geq 1.6\times 10^{12}T^{\frac{1}{2}}{\left(∆E\right)}^{3}$$

where ∆E represents the energy difference between the upper and lower levels of the spectral-line transition, which can be acquired from atomic spectra database of the National Institute of Standards and Technology (NIST).

**Figure 5:**
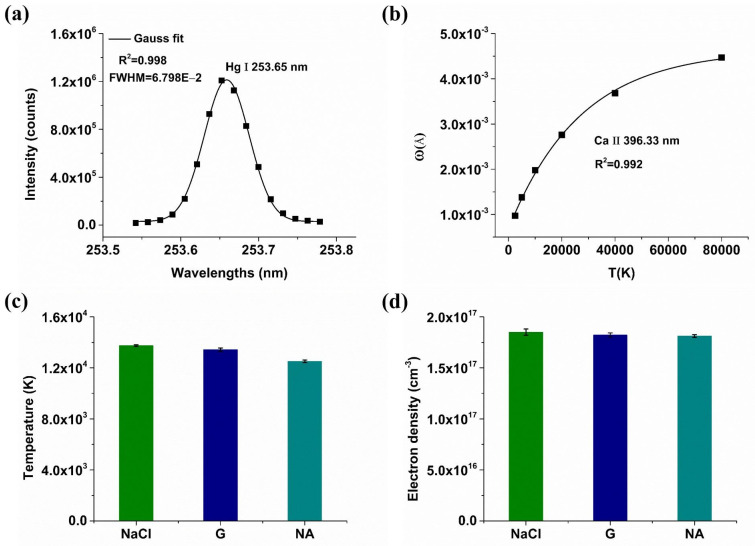
(**a**) Instrument-broadening fitting curve; (**b**) Collision coefficient versus plasma temperature curve; (**c**) Comparison of plasma temperature; (**d**) Comparison of electron density. G means graphite.

Abundant calcium (Ca) spectral lines in soil samples have necessitated the selection of six ionic lines for the determination of plasma temperature and electron density, with their parameters listed in Table 1. Electron density was calculated using Ca II 393.366 nm, and the Stark broadening result was obtained from Ca II 393.366 nm spectral-line broadening and instrument broadening measured via a standard low-pressure mercury lamp on Hg I 253.65 nm, as depicted in Figure 5a. The collision coefficient can be obtained from the curve between the plasma temperature and collision coefficient of Ca II 393.366 nm (Figure 5b), which was determined through the nonlinear regression of data presented in reference [37]. Figure 5c,d show the comparison results of the plasma temperature and electron density of soil samples with the optimal proportion of conductive material. With 10% NaCl, the average plasma temperature was 1.374 × 10^4^ K, and the average electron density was 1.850 × 10^17^ cm^−3^. With 20% graphite, the average plasma temperature was 1.343 × 10^4^ K, and the average electron density was 1.821 × 10^17^ cm^−3^. In the absence of conductive additives, the average plasma temperature was 1.251 × 10^4^ K, and the average electron density was 1.811 × 10^17^ cm^−3^. It is obvious that the plasma temperature and electron density slightly increased after adding conductive materials; especially when adding 10% NaCl, the plasma temperature and electron density were the highest. The results were consistent with the conclusions of spectral intensity and parameters. The high plasma temperature leaded to high spectral-line intensity and a large amount of sample ablation, thereby increasing the electron density. The results further confirmed that the addition of conductive materials lowered the ablation threshold and increased the coupling efficiency between the laser and the sample, leading to the increase in the plasma temperature and electron density. Compared with graphite, the ionization potential of chloride ions in NaCl is higher than that of carbon, and the melting point of NaCl is lower, resulting in higher ablation efficiency and higher plasma temperature and electron density after adding NaCl. In addition, according to Equation (6), the plasma was in the LTE state, and further quantitative analysis can be carried out.

### 2.3. Quantitative Determination of Pb Based on Conductive Materials

#### 2.3.1. Quantitative Analysis of Pb Based on Univariate Models

**Figure 6:**
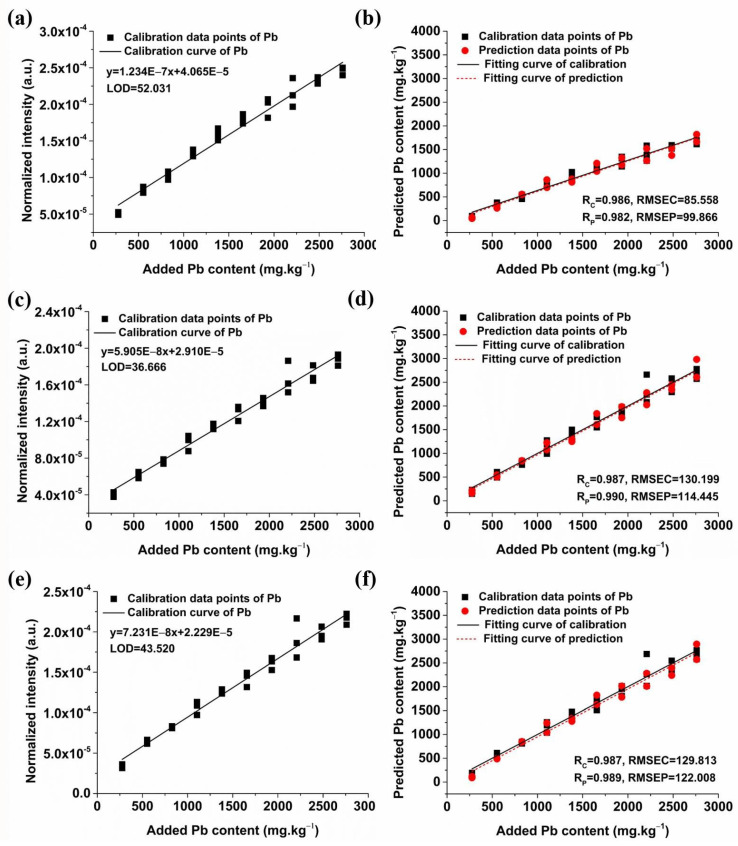
Univariate detection models and prediction results of soil Pb content with the optimal addition of NaCl based on the three primary characteristic lines of the Pb element. (**a**) The univariate model of Pb I 283.31 nm; (**b**) Prediction results of the univariate model based on Pb I 283.31 nm; (**c**) The univariate model of Pb I 368.35 nm; (**d**) Prediction results of the univariate model based on Pb I 368.35 nm; (**e**) The univariate model of Pb I 405.78 nm; (**f**) Prediction results of the univariate model based on Pb I 405.78 nm.

The soil samples were initially categorized into a calibration set (30 samples for each condition) and a predication set (20 samples for each condition). The calibration set is mainly used to build a model to reflect the relationship between the spectral intensity of the characteristic spectral line of Pb and added Pb content. The prediction set is mainly used to test the prediction ability of the model and to further evaluate the reliability and accuracy of the model based on the calibration set. Univariate models of peak intensity of three Pb spectral lines and Pb content were then established. Figure 6 shows the univariate model results of the Pb I 283.31, Pb I 368.35, and Pb I 405.78 nm lines of soil samples supplemented with 10% NaCl. Overall, the regression models for these spectral lines demonstrated satisfactory performance. The Rc of the univariate model of Pb I 283.31 nm was 0.986, with an RMSEC of 85.558, an Rp of 0.982, and an RMSEP of 99.866. The LOD of Pb content calculated based on this model was 52.031 mg/kg. It can be seen from Figure 6c,d that the Rc of the univariate model of Pb I 368.35 nm reached 0.987, with an RMSEC of 130.199, an Rp of 0.990, and an RMSEP of 114.445. The corresponding LOD from this model was 36.666 mg/kg. Figure 6e,f illustrate that the Rc for the univariate model of Pb I 405.78 nm was 0.987, with an RMSEC of 129.813, an Rp of 0.989, and an RMSEP of 122.008. The LOD determined by this model was 43.520 mg/kg. Comparatively, the models exhibit similar accuracy in both calibration and prediction sets for the three lead spectral lines. However, the LOD derived from the univariate model based on Pb I 368.35 nm was the most favorable.

**Figure 7:**
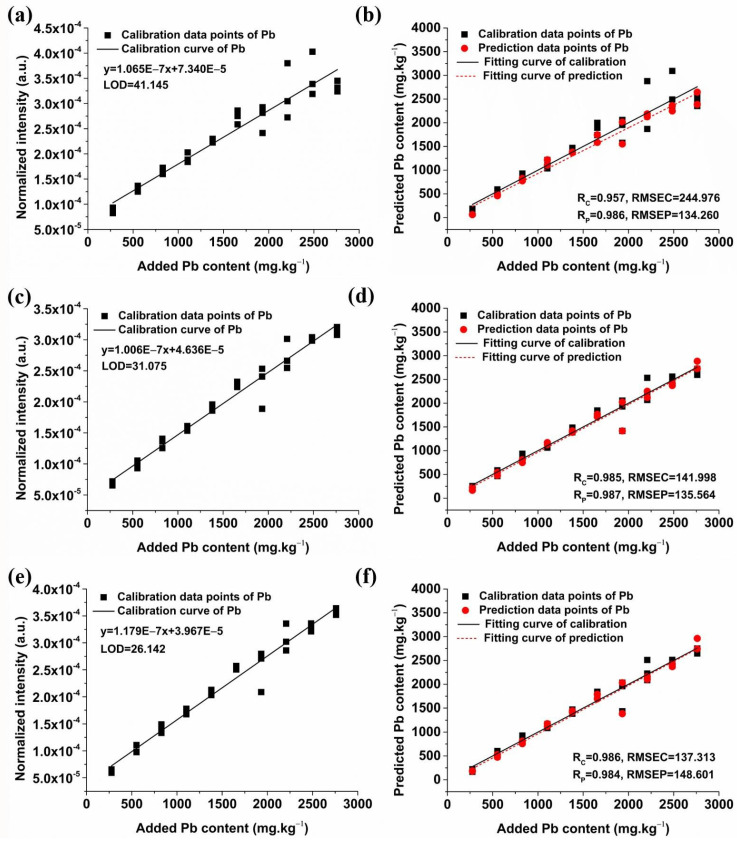
Univariate detection models and prediction results of soil Pb content with the optimal addition of graphite based on the three primary characteristic lines of the Pb element. (**a**) The univariate model of Pb I 283.31 nm; (**b**) Prediction results of the univariate model based on Pb I 283.31 nm; (**c**) The univariate model of Pb I 368.35 nm; (**d**) Prediction results of the univariate model based on Pb I 368.35 nm; (**e**) The univariate model of Pb I 405.78 nm; (**f**) Prediction results of the univariate model based on Pb I 405.78 nm.

Figure 7 shows the calibration and prediction results of univariate models for soil samples with 20% graphite. The univariate model of Pb I 283.31 nm demonstrated an Rc of 0.957, an RMSEC of 244.976, an Rp of 0.986, and an RMSEP of 134.260, with an LOD of 41.145 mg/kg. The model of Pb I 368.35 nm showed superior performance with an Rc of 0.985, an RMSEC of 141.998, an Rp of 0.987, an RMSEP of 135.564, and a lower LOD of 31.075 mg/kg. The model of Pb I 405.78 nm had an Rc of 0.986, an RMSEC of 137.313, an Rp of 0.984, and an RMSEP of 148.601, with an LOD of 26.142 mg/kg. In contrast, the prediction accuracy of the three univariate models was similar, but the LOD of the model based on the Pb I 405.78 nm was the lowest, which was different from the result of adding NaCl. Therefore, it is necessary to optimize the characteristic spectral lines for a more accurate detection of Pb content in soil in practical applications.

**Figure 8:**
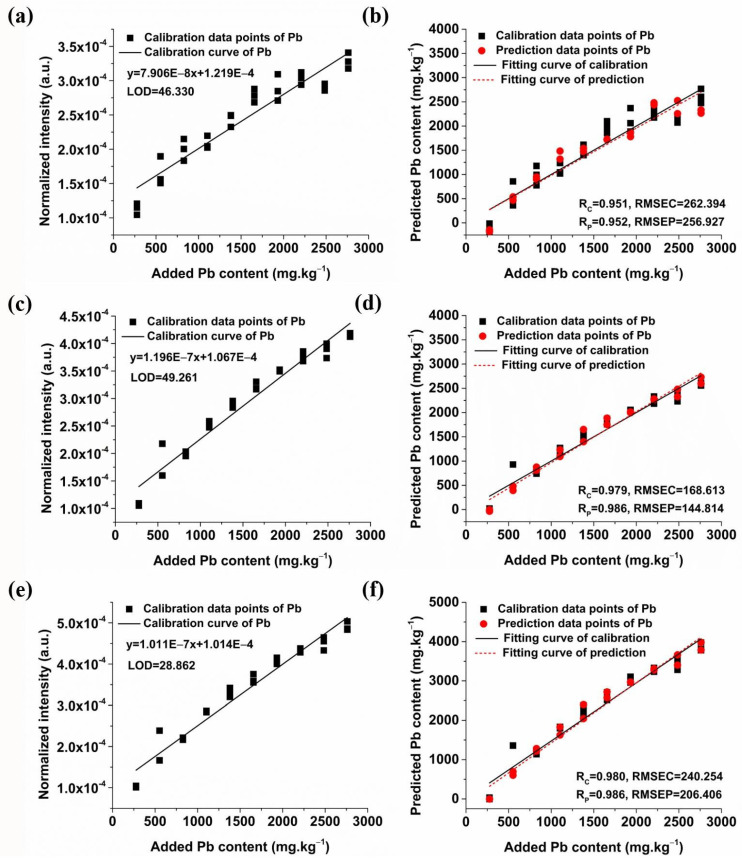
Univariate detection models and prediction results of soil Pb content without additives based on the three primary characteristic lines of the Pb element. (**a**) The univariate model of Pb I 283.31 nm; (**b**) Prediction results of the univariate model based on Pb I 283.31 nm; (**c**) The univariate model of Pb I 368.35 nm; (**d**) Prediction results of the univariate model based on Pb I 368.35 nm; (**e**) The univariate model of Pb I 405.78 nm; (**f**) Prediction results of the univariate model based on Pb I 405.78 nm.

Figure 8 shows the calibration and prediction results of univariate models for soil samples without conductive materials. The best prediction result was obtained by the univariate model of Pb I 405.78 nm, which demonstrated an Rc of 0.980, an RMSEC of 240.254, an Rp of 0.986, and an RMSEP of 206.406, with an LOD of 28.862 mg/kg. It is obvious that the predictive performance of the models was notably enhanced after adding conductive materials, as evidenced by the increase in Rp and decrease in LOD. In summary, the Pb I 405.78 nm univariate model with the addition of 20% graphite yielded the most favorable results, facilitating the accurate quantification of Pb in soil samples.

#### 2.3.2. Quantitative Analysis of Pb Based on Multivariate Models

Due to the intricate matrix effects inherent in soil and the abundance of spectral emission lines from various elements, univariate analysis may overlook critical information, thereby compromising the precision of quantitative assessments. In contrast, multivariate analysis techniques, such as PLSR, LS-SVM and ELM, are adept at mitigating the impact of matrix interferences and capitalizing on the full spectral information to enhance both the accuracy and the reproducibility of quantitative analysis. Consequently, these multivariate models were constructed using the entire collected spectral range to analyze the Pb content in soil samples with different additives, employing the same sample-partitioning strategy in the univariate models.

Table 2 summarizes the results of the PLSR, LS-SVM and ELM models for Pb content prediction in soil samples with different additives. With 10% NaCl, these models exhibited commendable predictive capabilities, with an Rc exceeding 0.991 and an Rp above 0.946. Specifically, the PLSR model achieved its optimal performance with an Rc of 0.991, an RMSEC of 104.477, an Rp of 0.966, and an RMSEP of 243.311, when the number of hidden layer variables was set to four. For the LS-SVM model, the optimal configuration was attained with a kernel function parameter (sig2) of 5.002 × 10^11^ and a regularization parameter (gam) of 1.361 × 10^10^, yielding an Rc of 1, an RMSEC of 3.30 × 10^−4^, an Rp of 0.946, and an RMSEP of 359.569. The ELM model demonstrated its best predictive efficacy with 15 hidden layer neurons, resulting in an Rc of 0.99, an RMSEC of 113.134, an Rp of 0.971, and an RMSEP of 350.716. The ELM model has the best prediction accuracy, which can realize the accurate quantitative detection of Pb in soil. With 20% graphite, the three types of models also showed good performance, with an Rc above 0.99 and an Rp above 0.95. When the hidden layer variable was four, the PLSR model has the best performance, with an Rc of 0.996, an RSMEC of 74.009, an Rp of 0.993, and an RMSEP of 108.609. When the sig2 and the gam of the LS-SVM model were 5.002 × 10^11^ and 1.361 × 10^10^, respectively, the model showed the best performance, with an Rc of 1, an RSMEC of 4.80 × 10^−4^, an Rp of 0.965, and an RMSEP of 447.346. The ELM model has the best prediction performance when the number of hidden layer neurons was 22, with an Rc of 0.994, an RSMEC of 85.441, an Rp of 0.954, and an RMSEP of 299.708. The PLSR model has the best prediction accuracy. Moreover, the optimal Rps for the three types of models were notably higher when conductive materials were incorporated. Generally, the models with graphite showed better prediction performance, which was consistent with the results of univariate analysis, suggesting that the addition of graphite significantly ameliorated the quantitative analysis of Pb in soil. Furthermore, among the univariate and multivariate models, the PLSR model, when augmented with graphite, exhibited the most superior predictive performance.

## 3. Materials and Methods

### 3.1. Soil Samples

Pb-contaminated soil samples were prepared by introducing the heavy metal into the certified reference material (CRM) of agricultural soil (GBW070046) purchased from the National Institute of Metrology, P. R. China. In accordance with the actual situation of Pb soil pollution in China, Pb(NO_3_)_2_ powder of 99% purity, obtained from Aladdin Reagent Shanghai Pure Biochemistry Technology Co., Ltd. (Shanghai, China), and deionized water were utilized to firstly prepare a series of Pb(NO_3_)_2_ solutions with different concentrations. Then, soil powders were incorporated into the prepared Pb(NO_3_)_2_ solutions, followed by thorough mixing with a magnetic stirrer. The mixture was dried in an oven at 60 °C for 8 h and then ground to obtain a uniform texture. Pb-contaminated soil samples with 10 distinct concentrations (276, 552, 828, 1104, 1380, 1656, 1932, 2208, 2484, and 2760 mg·kg^−1^) were successfully prepared. Soil powder with a Pb concentration of 1380 mg·kg^−1^ was mixed with NaCl crystals and graphite powder at mass fractions of 10%, 15%, 20%, 25%, and 30%, respectively, followed by grinding and tableting to fabricate soil tablets with varying contents of additive materials for optimizing the addition ratio of conductive materials. The soil powders with 10 distinct Pb concentrations were then mixed with the optimal proportions of NaCl and graphite. Then, 0.3 g mixture was taken for tableting at a pressure of 10 MPa for 30 s (FY-24, SCJS, Tianjin, China), and circular soil tablets with a diameter of 12 mm and thickness of 2 mm were obtained. Three soil tablets for each concentration of Pb were prepared. Finally, 15 samples were obtained for the optimization of NaCl and graphite content, respectively, and 50 soil samples individually supplemented with NaCl and graphite were obtained for the quantitative detection of Pb.

### 3.2. Spectral Acquisition

**Figure 9:**
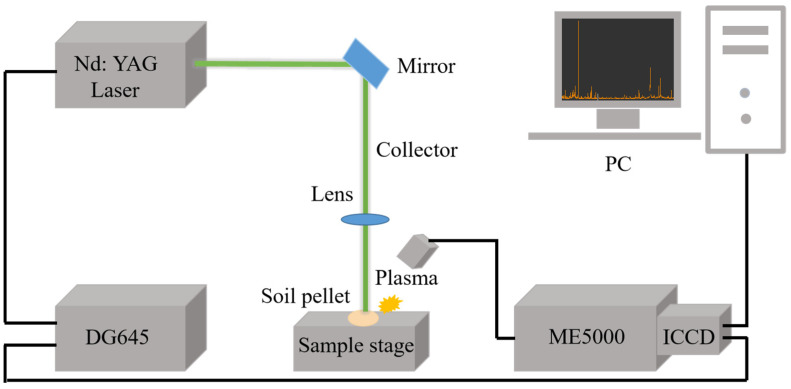
Schematic diagram of LIBS system.

The LIBS system used in the experiment mainly consists of a Q-switched Nd:YAG pulsed laser (Vlite-200, Beamtech Optronics, Beijing, China) for sample ablation to produce plasma, operating at 532 nm with a pulse energy of up to 200 mJ, with an 8 ns pulse duration and a repetition frequency of 1 to 10 Hz; an Echelle grating spectrometer (SR-500i-A-R, Andor Technology, Belfast, UK); an intensified charge-coupled device (ICCD) camera (iStar DH340T, Andor Technology, Belfast, UK) for the detection of spectral signals; and a digital delay generator (DG645, Stanford Research Systems, US) for controlling the delay time between the operation of the laser and the ICCD camera. Specifically, the spectrometer functions to disperse the signals generated by plasma, while the detector converts the dispersed optical signals into electrical signals, which are then recorded and analyzed by a computer. Moreover, an optical path system with lenses and mirrors, a X-Y-Z sample stage, a signal collector, a computer, and control software were also used. Additionally, a mercury-argon lamp (HG-1, Ocean Optics, USA) and a deuterium-halogen light source (DH-2000-BAL-CAL, Ocean Optics, USA) were employed for wavelength and intensity calibration, respectively. Figure 9 shows the schematic diagram of the LIBS system. The experimental parameters were optimized with a delay time of 4 μs and a laser energy of 110 mJ. The distance from the lens to the sample was 98 mm (the focal length of the len was 100 mm) and the gain was set to 2000. Finally, soil tablets were positioned on the X-Y-Z stage, with the laser beam was precisely aligned 2 mm beneath the sample surface to acquire LIBS spectra. To mitigate shot-to-shot variability and ensure robust data acquisition, spectra of 16 distinct spots were obtained by the movement of stages, with a movement path of 4×4 in steps of 1 mm. Spectral signals with five-times accumulation at each spot were acquired, and the mean of 80 spectra was used as the representative LIBS spectrum for each soil tablet.

### 3.3. Data Analysis

#### 3.3.1. Data Preprocessing

Due to the characteristics of the soil samples (uniformity, surface flatness and its matrix effect), differences in instrument performance and environment will generate random noise and abnormal spectra, affecting the stability of the target signal. Thus, the obtained LIBS spectra were firstly preprocessed using wavelet transform (WT), area normalization and outlier rejection to reduce the impact of the above factors on the signal. WT is a prevalent denoising method that decomposes the original spectrum into a low-frequency signal containing spectral features and a high-frequency signal with noise using wavelet functions, and noise reduction can be achieved by setting a threshold to eliminate the high-frequency noise while retaining the low-frequency signal. The denoising effectiveness varies with the level of wavelet decomposition, and the optimal level is determined when the maximal signal-to-noise ratio (SNR) or the minimal root-mean-square error (RMSE) is obtained [39]. Daubechies 5 with decomposition scale 3 was used in this study. Moreover, studies have shown that the normalization of LIBS data can effectively reduce the sample matrix effect and point-to-point fluctuations and improve the stability of spectral signals [40,41]. Area normalization is a common method in LIBS data analysis, which divides the spectral value of each spectral line by the integration of the overall spectral background and spectral intensity area of the sample to obtain the area-normalized spectrum [42]. Additionally, outlier rejection is a commonly used method in LIBS data analysis to remove spectra with large deviations from the original signal, generated when the plasma makes random transitions according to the energy-level difference. The median absolute deviation (MAD) was used to eliminate the abnormal spectra [43,44]. The peak intensity of Pb I 405.78 nm was selected as the criterion for outlier identification, as the spectral line of Pb I 405.78 nm exhibited better stability among the three Pb spectral lines. Specifically, the median and MAD of peak intensity of Pb I 405.78 nm were calculated, and spectra with an intensity deviation from the median of Pb I 405.78 exceeding 2.5 times the MAD were eliminated until there were no outliers or the remaining spectra constituted less than 75% of the raw spectra.

#### 3.3.2. Quantitative Analysis Methods

Univariate analysis is a fundamental technique in LIBS quantitative analysis. Under ideal conditions, the spectral intensity is directly proportional to the elemental concentration in the sample. Consequently, the calibration curve of the element can be established based on the intensity of the LIBS spectrum and the concentration of the corresponding element. The concentration of unknown samples can be inferred from their spectral intensities based on the calibration curve [45]. In addition, the univariate model performance is usually evaluated using R—root-mean-square error (RMSE). The R indicates the precision of the model’s quantitative analysis, with values closer to one signifying higher accuracy. In this study, the correlation coefficients for the calibration and prediction sets were denoted as RC and RP, respectively. The RMSE quantifies the standard deviation of the model’s predictions from the actual chemical values, reflecting the model’s predictive error. The closer the RMSE value is to zero, the more accurate and reliable the model’s prediction is. For this study, the RMSEs for the calibration and prediction sets were represented as RMSEC and RMSEP, respectively. Also, the LOD is a critical parameter for assessing analytical performance. As defined by the International Union of Pure and Applied Chemistry (IUPAC), the LOD is the lowest concentration obtained from the minimum analytical signal that can reasonably be detected by a specific detection method, and a lower LOD close to zero indicates superior detection performance. The calculation method of LOD can be obtained in the reference.

Partial least-squares regression (PLSR) is a multivariate linear analysis technique widely used for the quantitative analysis of spectral data [46,47]. Specifically, this method decomposes the original data to obtain linear and mutually independent latent variables through linear transformation. In practice, further analysis is conducted on only the first few latent variables that encapsulate most of the information of the original variables [48]. Given the risk of overfitting when the number of samples is less than the number of variables, full cross-validation was used to correct the model, and the latent variable corresponding to the minimum root-mean-square error of cross-validation (RMSECV) was determined as the optimal latent variable [49]. Additionally, the PLSR model was assessed using the same evaluation indicators as the univariate model.

Least-squares support vector machine (LS-SVM) is an optimization statistical learning method based on support vector machines (SVMs). Known for its strong generalization capabilities, it is extensively applied in both linear and nonlinear quantitative analysis. The method involves mapping the original data into a high-dimensional space via a nonlinear transformation, followed by performing linear regression in this space using a linear hyperplane to derive the fitting function [50,51]. In this study, a radial basis function (RBF) kernel was employed to construct the model, and optimal parameters of sig2 and gam were determined through a grid-search procedure within the range of 103 to 1010, which corresponded to the minimum root-mean-square error of cross-validation (RMSECV). The evaluation indicators of the LS-SVM model were the same as those of the univariate model.

The extreme learning machine (ELM) is a feedforward neural network that leverages random feature mapping. The essence of the ELM lies in the random initialization of the weights and biases from the input to the hidden layer, followed by the determination of the output layer weights through least-squares or other optimization algorithms. This approach significantly reduces training time while maintaining robust generalization capabilities [52,53]. A pivotal advantage of the ELM is its swift training velocity and reduced computational complexity, making it highly effective for a variety of applications, including data mining, pattern recognition, and quantitative prediction. In this study, the ELM method was utilized for quantitative analysis, where the response variable was the actual reference concentration of the element. During model operation, the number of hidden layer nodes started from one and gradually increased in intervals of one until it equaled the number of the calibration set, and the optimal ELM model and number of hidden layer nodes corresponded to the minimum RMSE of the prediction set.

### 3.4. Software Tools

To design experiments and optimize experimental parameters, Design Expert (ver.8.05, CAMO AS, Oslo, Norway) was used. LIBS spectra were collected using Andor SOLIS for Imaging (v4.26, Andor Technology, UK). Unscrambler X 10.1 (CAMO, Process, AS, OSLO, Norway) and MATLAB R2009a (v7.8, The MathWorks, Inc., Natick, MA, USA) were used for data processing and quantitative analysis. VK-K Series was used for the analysis of the three-dimensional profile data of ablation craters. In addition, graphics were created using Origin Pro 8.0 SR0 (Origin Lab Corporation, Northampton, MA, USA).

## 4. Conclusions

This study revealed that LIBS combined with sample pretreatment based on conductive materials of NaCl and graphite and chemometrics methods had a good ability to determine Pb content in soil. Under the optimal addition of NaCl and graphite, univariate and multivariate models showed the good prediction performance of Pb content in soil. At the addition levels of 10% NaCl and 20% graphite, the three spectral lines of Pb exhibited enhanced intensity and favorable spectral parameters. The incorporation of conductive materials lowered the ablation threshold, enhanced the laser-sample coupling efficiency, and elevated the amount of ablation, the electron density and the plasma temperature. For quantitative detection, adding graphite showed a stronger improvement of spectral sensitivity, and the PLSR model with the optimal amount of graphite had better prediction performance, with an Rp that reached 0.994. In addition, among the three spectral lines of Pb, the univariate model of Pb I 405.78 nm showed the best prediction performance, with an Rp of 0.984 and the lowest LOD of 26.142 mg/kg. Adding conductive materials, especially graphite, can improve the stability of Pb spectral signals and the prediction accuracy of the quantitative model. The method obtained comparable results to other signal-enhancement methods currently in use, such as dual-pulse excitation, spatial confinement, magnetic confinement and inert gas enhancement, but with lower costs, which provides a new direction for the accurate quantitative detection of heavy metals in soil. In general, improving the spectral intensity can improve the quantitative analysis accuracy to a certain extent, but improving the sensitivity and stability of spectral lines is more critical. In future research on signal-enhancement methods for the quantitative detection of heavy metals, spectral intensity and the parameters of SBR, SNR, and RSD need to be considered, and the accuracy and reliability of quantitative detection based on various chemometric methods are the most important factors. Moreover, the mechanism of signal enhancement should be explored based on the analysis of the three-dimensional profile data of ablation craters and plasma parameters (plasma, temperature and electron density). 

## Figures and Tables

**Table 1:** Spectral parameters of calcium (Ca).

Spectral Line (nm)	Spontaneous Transition Probability *A_ki_* (10^8^ s^−1^)	Excitation Energy of Upper Level *E_k_* (ev)	Statistical Weight of Upper Level *g_k_*
Ca II 315.887	3.10	7.047168	2
Ca II 317.933	3.60	7.049550	4
Ca II 370.603	0.88	6.467875	2
Ca II 373.731	1.70	6.467875	2
Ca II 393.366	1.47	3.150984	2
Ca II 386.847	1.40	3.123349	2

**Table 2:** Results of multivariate detection model for soil Pb content with different conductive materials.

Additive	Model	Parameter	Calibration		Prediction	
			RC	RMSEC	RP	RMSEP
NaCl	PLSR	4	0.991	104.477	0.966	243.311
	LS-SVM	(5.002 × 10^11^, 1.361 × 10^10^)	1.000	3.300 × 10^−4^	0.946	359.569
	ELM	15	0.990	113.134	0.971	350.716
	PLSR	5	0.996	74.009	0.993	108.609
graphite	LS-SVM	(5.002 × 10^11^, 1.361 × 10^10^)	1.000	4.800 × 10^−4^	0.965	447.346
	ELM	22	0.994	85.441	0.954	299.708
NA	PLSR	3	0.985	136.911	0.978	179.760
	LS-SVM	(5.002 × 10^11^, 1.361 × 10^10^)	1.000	4.442 × 10^−4^	0.880	433.261
	ELM	21	0.992	100.431	0.968	209.882

## Data Availability

Data will be available on request.
